# Military-related mild traumatic brain injury: clinical characteristics, advanced neuroimaging, and molecular mechanisms

**DOI:** 10.1038/s41398-023-02569-1

**Published:** 2023-08-31

**Authors:** Sharon Y. Kim, Ping-Hong Yeh, John M. Ollinger, Herman D. Morris, Maureen N. Hood, Vincent B. Ho, Kwang H. Choi

**Affiliations:** 1 School of Medicine, Uniformed Services University, Bethesda, MD USA; 2 Program in Neuroscience, Uniformed Services University, Bethesda, MD USA; 3 National Intrepid Center of Excellence, Walter Reed National Military Medical Center, Bethesda, MD USA; 4 Department of Radiology and Radiological Sciences, Uniformed Services University, Bethesda, MD USA; 5 Department of Radiology, Walter Reed National Military Medical Center, Bethesda, MD USA; 6 Center for the Study of Traumatic Stress, Uniformed Services University, Bethesda, MD USA; 7 Department of Psychiatry, Uniformed Services University, Bethesda, MD USA

**Keywords:** Diagnostic markers, Molecular neuroscience

## Abstract

Mild traumatic brain injury (mTBI) is a significant health burden among military service members. Although mTBI was once considered relatively benign compared to more severe TBIs, a growing body of evidence has demonstrated the devastating neurological consequences of mTBI, including chronic post-concussion symptoms and deficits in cognition, memory, sleep, vision, and hearing. The discovery of reliable biomarkers for mTBI has been challenging due to under-reporting and heterogeneity of military-related mTBI, unpredictability of pathological changes, and delay of post-injury clinical evaluations. Moreover, compared to more severe TBI, mTBI is especially difficult to diagnose due to the lack of overt clinical neuroimaging findings. Yet, advanced neuroimaging techniques using magnetic resonance imaging (MRI) hold promise in detecting microstructural aberrations following mTBI. Using different pulse sequences, MRI enables the evaluation of different tissue characteristics without risks associated with ionizing radiation inherent to other imaging modalities, such as X-ray-based studies or computerized tomography (CT). Accordingly, considering the high morbidity of mTBI in military populations, debilitating post-injury symptoms, and lack of robust neuroimaging biomarkers, this review (1) summarizes the nature and mechanisms of mTBI in military settings, (2) describes clinical characteristics of military-related mTBI and associated comorbidities, such as post-traumatic stress disorder (PTSD), (3) highlights advanced neuroimaging techniques used to study mTBI and the molecular mechanisms that can be inferred, and (4) discusses emerging frontiers in advanced neuroimaging for mTBI. We encourage multi-modal approaches combining neuropsychiatric, blood-based, and genetic data as well as the discovery and employment of new imaging techniques with big data analytics that enable accurate detection of post-injury pathologic aberrations related to tissue microstructure, glymphatic function, and neurodegeneration. Ultimately, this review provides a foundational overview of military-related mTBI and advanced neuroimaging techniques that merit further study for mTBI diagnosis, prognosis, and treatment monitoring.

## Introduction

Traumatic brain injury (TBI) is defined as a disruption of normal brain function caused by a bump, blow, jolt, or penetrating head injury [1, 2]. Military service members (SMs) are at increased risk of TBI from falls, car accidents, strikes, or explosions in combat or operational settings. According to the Defense and Veterans Brain Injury Center (DVBIC), more than 450,000 TBIs among U.S. SMs worldwide have been reported between 2000 and 2022, with over 80% of them classified as mild (mTBI) [3].

Per Department of Defense (DoD) TBI guidelines, the severity of TBI is assessed through various criteria, including neuroimaging, the Glasgow Coma Scale, and duration of loss of consciousness (LOC), alteration of consciousness (AOC), and posttraumatic amnesia (PTA) (Table 1) [4]. However, mTBI is especially difficult to diagnose due to its highly heterogeneous nature and lack of overt clinical neuroimaging findings. Although mTBI was once considered benign and noncritical compared to more severe TBIs, a growing body of evidence has demonstrated the neuropsychiatric consequences of mTBI, including chronic post-concussion symptoms, pain and headaches, cognition, memory, mood, sleep, vision, and hearing [4–6].

**Table 1:** Classification of TBI Severity based on VA/DoD Guidelines for TBI.

Criteria	Mild TBI	Moderate TBI	Severe TBI
Structural imaging	Normal	Normal or abnormal	Normal or abnormal
Loss of Consciousness (LOC)	0–30 min	>30 min and <24 h	>24 h
Alteration of consciousness (AOC)	up to 24 h	>24 h	>24 h
Posttraumatic amnesia (PTA)	0-1 day	>1 and <7 days	>7 days
Glasgow Coma Scale (GCS)	13–15	9–12	<9

Considering the high morbidity of mTBI in military populations, lack of robust neuroimaging biomarkers, and debilitating post-injury symptoms of mTBI, this review attempts to (1) summarize the nature and mechanism of mTBI in military combat settings, (2) describe clinical characteristics of mTBI and associated comorbidities, such as post-traumatic stress disorder (PTSD), (3) highlight advanced neuroimaging techniques used to study mTBI and the molecular mechanisms that can be inferred, and (4) discuss future directions of advanced neuroimaging research. However, this is not a comprehensive review of all existing literature on military-related mTBI, and several reviews have already been published. [7–10] Thus, prior reviews should complement this work. Specifically, we describe various neuroimaging techniques used to study military-related mTBI and review recent work in each neuroimaging domain.

## Etiological mechanisms of military-related mTBI

The injury mechanisms of mTBI have been reviewed in detail elsewhere [9, 11]. Briefly, the two main injury types of deployment-related mTBI are non-blast (or blunt) and blast injuries. Blunt head injuries can occur from accidents, falls, or violent impacts. By contrast, blast injuries are more specific to the military where mTBIs are elicited from shock waves induced by explosive weapons, including improvised explosive devices (IED) and heavy munitions firing [12]. The DoD has classified the mechanisms of blast-related injuries into five categories (Table 2) [12–14]. Blast injury is the most common injury mechanism in military TBI, accounting for approximately 60% of all military TBI, and as much as 80% of mTBI [15–18]. Due to recent advances in protective body armor, helmet design, battlefield medical protocol, and medical evacuation strategies, more SMs survive battlefield injuries that were otherwise fatal in past conflicts. Thus, a larger proportion of SMs are returning home with polytrauma injuries, including mTBIs and blast-related impairments [17, 19–21].

**Table 2:** Mechanisms of blast-related TBI based on DoD Blast Injury Research Program (from DoDD 6025.21E).

Injury Type	Mechanism	Pathology
Primary	Injuries caused by blast overpressure from high explosives. Injuries due to blast wave air-soft tissue interface in the body	Non-impact induced mTBI; tympanic membrane rupture; middle ear damage pulmonary barotrauma; gastrointestinal tract rupture or hemorrhage; eye rupture
Secondary	Injuries caused by flying fragments, debris, or objects caused by the blast	Penetrating injuries affecting any part of the body
Tertiary	Injuries caused by individuals being knocked over by the blast wave, causing impact with surrounding objects or ground; contrecoup injuries	Fractures/amputations of limbs; open/closed brain injury; blunt injury; compartment syndrome; crush injury
Quaternary	All other explosion-related injuries, illnesses, or diseases caused by the blast but not due to primary, secondary, or tertiary mechanisms	Burns; exacerbation of respiratory disease; angina, myocardial infarction; crush injuries
Quinary	Injuries related to the clinical consequences of post-detonation environmental contaminant, including chemical (i.e., sarin or chlorine), biological (i.e., viruses or bacteria), and radiological (i.e., dirty bombs) substances	Illnesses, injuries, or diseases caused by chemical, biological, or radiological substances
Psychological trauma	Psychological trauma that can develop following a blast-related concussion	Post-traumatic stress disorder

Studies have utilized human and animal head modeling as well as computational methods to characterize the neurological, pathological, and molecular consequences of blast-related injury [22–28]. Briefly, pressurization changes of the brain caused by shock waves can cause strain and shearing of brain tissue, blood vessels, and neurons that may be accompanied by contusions, hemorrhaging, and diffuse axonal injury. Inflammatory-related abnormalities in brain tissue and edema [29] can also result in a range of neuropsychiatric symptoms, including but not limited to headaches, dizziness, nausea, and AOC [23]. Further, a blast wave can lead to a “coup-contrecoup” injury, in which the head is suddenly accelerated and decelerated due to blast pressures that cause alternating anterior-posterior impacts of the brain within the skull [27]. Other consequences of intense force on the brain can include harm to axons and microvessels [30], disturbances in ion concentrations inside and outside brain tissue cells [31], an accelerated rate of glucose metabolism in neurons [32], and compromised integrity of the blood-brain barrier (BBB) resulting in poor perfusion of local brain functional areas [30].

Following the initial brain tissue or axonal injury, subsequent mechanisms of injury unfold, involving biochemical, metabolic, and cellular alterations that occur in the time frame of minutes, days and months [33–35]. While some aspects of these biological processes can result in macroscopic changes visible in standard imaging techniques, including those related to inflammation, microvascular damage, and neuroplasticity [36], many changes occur at a much smaller spatial scale that cannot be detected through conventional imaging modalities. Notably, a recent animal study showed that exposure to double blast waves has significant effects on various markers associated with synaptic function, glymphatic system, myelin, neuronal health, and neurovascular function [28]. However, such alterations were not accompanied by changes in behavior, supporting the hypothesis that an asymptomatic altered status can be caused by repeated blast exposures.

It is important to note that blast exposure affects various organ systems, including nervous, pulmonary, gastrointestinal, cardiovascular, and endocrine systems [37]. The damage to the body resulting from blast-related injuries can subsequently affect the brain through various mechanisms. For example, the body’s response to blast-related injuries can trigger a systemic inflammatory response, which can affect the brain through the release of pro-inflammatory cytokines and other mediators. This neuroinflammation can contribute to secondary brain damage and neurological symptoms. Moreover, lung damage or significant blood loss can lead to decreased oxygen supply (hypoxia) and inadequate blood flow (ischemia), which can lead to neuronal cell death and cognitive impairments. Finally, blast-related injuries can disrupt the body’s metabolic and electrolyte balance, impacting brain function and contributing to neurological complications.

## Diagnosis of military-related mTBI

The DoD criteria for diagnosing mTBI are outlined in Table 1. Military mTBI can be challenging to diagnose due to the concealment of mTBI, unpredictability of pathological changes, and delay of post-injury clinical evaluations [38]. Eyewitness and casualty self-reports are usually the only available information used to diagnose military mTBI in urgent settings of the battlefield environment where no trained healthcare personnel is available. The blast itself may result in LOC in the patient and potential eyewitnesses further complicating any attempt to reconstruct the actual head injury mechanism(s) sustained by the subject, making it difficult for clinicians to gather accurate information and assess for mTBIs post-injury [39]. Further, reliable estimates of the burden of blast-related mTBI are lacking due to the ambiguity of a precise clinical definition, absence of objective tests for diagnosis of blast-related mTBI in the battlefield, and the potential overlap with other conditions such as PTSD. However, there are ongoing efforts to enable the more precise identification of injury type and mechanisms on the battlefield through advanced operational equipment, including blast pressure sensors on helmets and cameras on uniforms that can visually capture the surrounding environment [40]. Better understanding of blast-related head injuries will enable the development of more advanced protective head equipment that can be widely employed in military contexts [41].

**Fig. 1:**
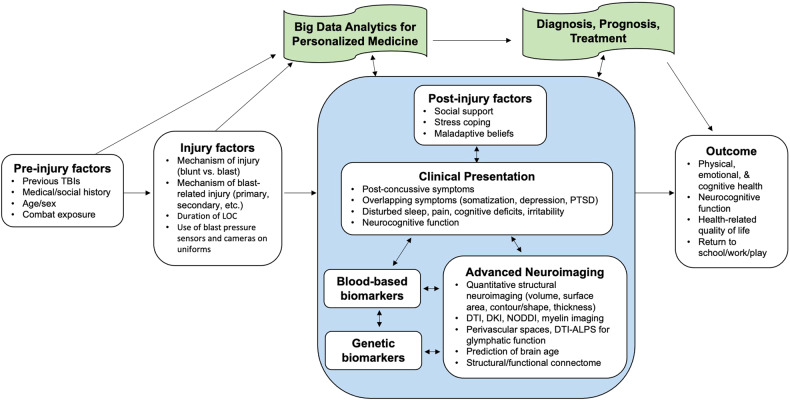
A conceptual framework for the study of military-related mTBI. Pre-injury and injury factors influence the clinical presentation of mTBI patients. In military settings, prior combat exposure and history of TBI influence post-injury clinical presentation and outcome. Important injury factors in military combat settings include the mechanism of injury (blast vs. blunt), type of blast-related injury (refer to Table 2), duration of LOC, and use of uniform gear (blast pressure sensors, cameras). Big data analytics of post-injury factors, clinical symptoms, blood-based biomarkers, genetic biomarkers, and advanced neuroimaging enable a more personalized medicine approach for the proper diagnosis, prognosis, and treatment of military-related mTBI. A culmination of various factors and multi-modal diagnostic, prognostic, and treatment approaches can influence post-injury outcomes. Figure inspired by and adapted from Polinder et al. [193].

Figure 1 describes current topics in research on military-related mTBI. Indeed, military SMs and civilians often experience different circumstances and mechanisms of injury that lead to mTBI. Understanding the similarities and differences in these populations can help identify specific risk factors, injury patterns, clinical characteristics, and long-term sequelae that may be unique to each population, overall improving care and optimizing outcomes.

## Clinical characteristics of military-related mTBI

The onset of mTBI-related clinical symptoms can manifest at different time points divided into acute, subacute, and chronic phases. Within the first fifteen minutes post-injury, there is a significant decline in neuropsychological performance [42], and such deficits can linger for a week or longer [43]. Neuropsychiatric sequelae of mTBI, including cognitive impairment, major depression, anxiety, neuroendocrine disorders, and sleep disorders, may occur within a few months post-injury. These symptoms can overlap with post-concussion syndrome (PCS), which affects a substantial proportion of mTBI patients (3–53%) [44, 45]. Specifically, PCS is challenging to diagnose as its symptoms are non-specific and similar to other disorders, including major depression [46, 47], chronic pain [48], PTSD [49, 50] somatic symptom disorder [51], and substance use disorders [52], all of which are commonly seen following mTBI [38]. Further, even when the mTBI shows no imaging abnormalities and is thought to be clinically cured, such neuropsychiatric symptoms can still persist, affecting the physical and mental well-being of SMs [53]. Neuropsychological assessments in the chronic stages of mTBI (even on the time scale of months) have also been criticized as non-specific and insensitive. [54, 55] Namely, several studies have raised questions regarding the validity of these evaluations and whether they accurately reflect real-world functioning [56–58].

Finally, studies have shown that military-related mTBI, including blast-related head injuries, is associated with chronic neurodegenerative changes, namely chronic traumatic encephalopathy (CTE) [59]. CTE is an advancing condition marked by identifiable tangles of tau proteins (also known as neurofibrillary tangles [NFTs]) and sometimes oligomers of transactive response DNA binding protein 43 (TDP43). These tangles and oligomers tend to occur in specific areas near reactive astrocytes and microglia, particularly in the perivascular and subcortical regions. CTE is currently only diagnosed postmortem by neuropathological identification of NFTs. Thus, there is a need for improved multi-modal diagnostic approaches, combining neuroimaging, blood/cerebrospinal fluid (CSF) analysis, and neuropsychological tests, for detecting and treating post-injury neurodegenerative sequalae (Fig. 1).

## Review of neuroimaging techniques

This review focuses primarily on magnetic resonance imaging (MRI) rather than other imaging techniques including electroencephalogram (EEG), magnetoencephalography (MEG), and positron emission tomography (PET), which have been discussed in other review articles [7, 60, 61]. Each imaging modality discussed here has its own advantages and disadvantages in probing particular aspects of brain structure and function. Thus, potential molecular mechanisms and biological processes that can be inferred from neuroimaging will be also discussed.

### Structural MRI

Structural imaging utilizes contrasts to visualize anatomical properties of the brain. However, routine structural MRI findings are frequently normal following mTBI and have a limited role in diagnosis and management. Thus, advanced quantitative techniques are important in measuring more subtle alterations, including those related to white matter (WM) hyperintensities, volumetry (amount of brain tissue in different regions), and morphometry (shape of anatomic brain regions).

**Fig. 2:**
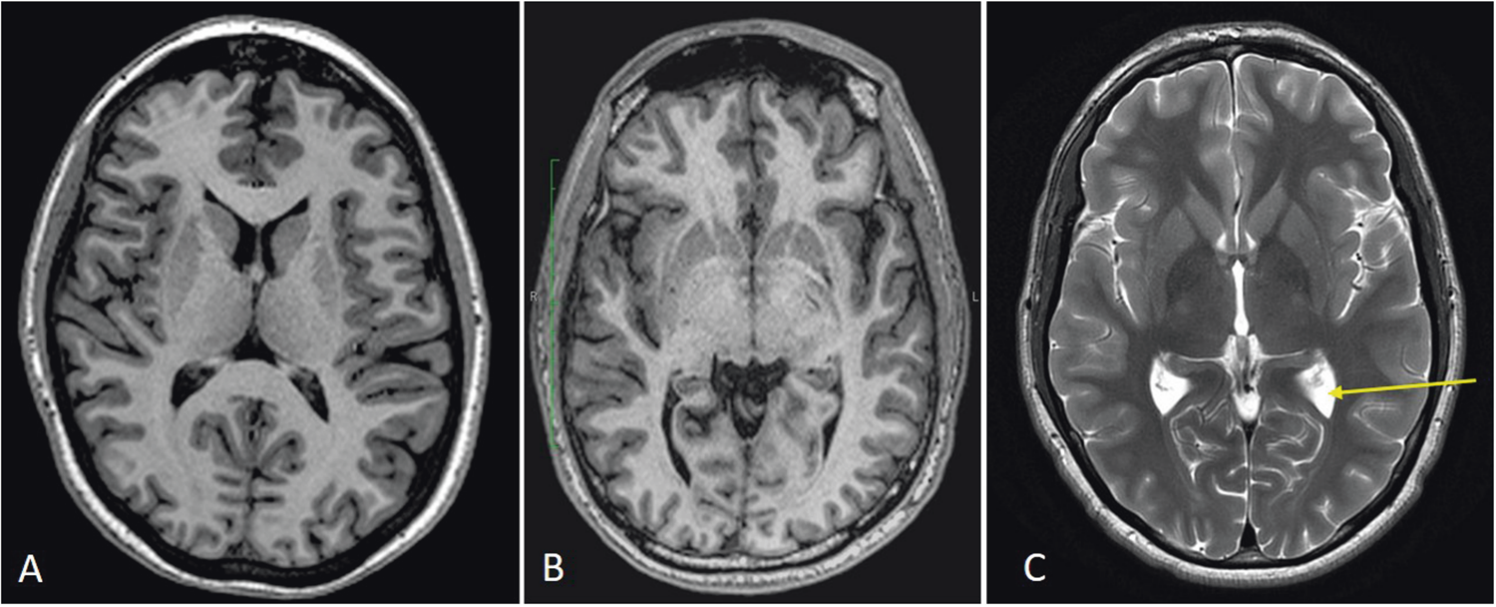
Structural MRI. T1 (**A**, **B**) and T2 (**C**) weighted images are two main types of image contrast used to characterize tissue and structures in MRI. A and B show images from a T1w MPRAGE sequence. **A** is a traditional T1w MPRAGE that clearly delineates the white and gray matter structures in the brain as shown in this axial image. White matter is brighter than gray matter on T1 weighted images. **B** is produced from the newer MPRAGE PROMO (PROspective MOtion correction) sequence, which provides the utility of reducing motion artefacts which can be problematic in some patients. **C** is produced from the T2w fast spin echo sequence that complements the T1w images. Fluid is bright on T2w images as demonstrated by the bright CSF in the ventricles (arrow). Gray and white matter are reversed with white matter being darker on T2w images.

**Fig. 3:**
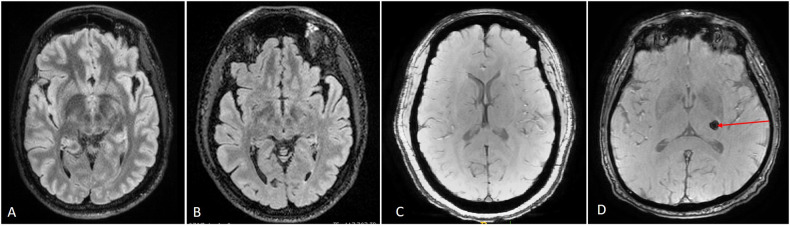
T2 FLAIR and SWI images. T2 FLAIR is a technique that accentuates the white matter hyper-intensities while nulling the signal from CSF (**A**, **B**). On T2 FLAIR sequences, the white matter is dark, the gray matter is bright, and the CSF in the ventricles is dark. This technique allows for subtle white matter hyperintensities to be detectable even in areas close to the ventricles. Susceptibility-weighted imaging (SWI) is a gradient echo technique that takes advantage of both phase and magnitude effects to accentuate the presence of ferromagnetic, paramagnetic, and diamagnetic compounds (**C**, **D**). Thus, SWI is used to identify microbleeds, blood products, and calcium. **C** is a standard SWI image and **D**. is from a patient with a small hemorrhage (red arrow) that is seen as a dark ring.

One of the benefits of MRI is the ability to perform a variety of pulse sequences to evaluate different tissue characteristics during the same exam period without risks associated with ionizing radiation inherent to X-ray-based imaging, such as computerized tomography (CT). T1-weighted (T1w) MRI pulse sequences are primarily used to delineate anatomy (Fig. 2A, B). T1w images differentiate gray matter (GM) from WM, which allows for cortical surface modeling and measurement of cortical thickness. By contrast, T2-weighted (T2w) MRI pulse sequences are used to identify increased fluid content and help to define areas of abnormalities such as edema (Fig. 2C). A few additional sequences have been created to depict specific structural abnormalities, including fluid-attenuated inversion recovery (FLAIR) and susceptibility weighted imaging (SWI) / quantitative susceptibility maps (QSM), commonly used to identify WM hyperintensities (WMHI) and microbleeds in mTBI, respectively (Fig. 3). Aberrations in structural brain imaging can represent brain abnormalities and pathological processes following mTBI. These sequences provide a variety of tissue contrast types to help clinicians characterize brain pathology.

### Volumetry

Studies to date have reported both global and regional GM and WM volumetric atrophy following mTBI, even several years post-injury [62–65]. Some studies have also examined changes specific to the cortical surface, including cortical thickness, which reflects underlying regional GM integrity and is hypothesized to be geometrically related to both cortical surface area and volume [66, 67]. A common observation in mTBI is cortical thinning or reduced GM volume, due to neurodegenerative processes, including cell death or loss of dendritic branches [68, 69]. Patel et al. [70] recently reported volumetric loss in several GM, WM, subcortical GM, and parenchymal regions in a mTBI military cohort compared to a non-brain-injured military cohort. Santhanam and colleagues [71] also identified age-related patterns of cortical thinning in active-duty SMs and Veterans (SMVs) with a history of mTBI. Specifically, the effect of mTBI diagnosis and age on cortical thickness (group x age interaction) was found to be significant for many brain regions, including bilateral parietal and left frontal and temporal cortices. Together, these studies suggest that the presence of mTBI is associated with age-related cortical thinning across the cortex in military populations.

Due to notable comorbidity between PTSD and mTBI in military populations, it can be challenging to study the association between mTBI and brain volume without considering confounding effects of PTSD. Yet, it is crucial to identify to what extent each condition is associated with brain volumes and how they may interact to influence brain pathology. Martindale et al. found that deployment-related mTBI was associated with lower bilateral hippocampal volume and right medial orbitofrontal cortex volume [72]. However, neither current nor lifetime PTSD diagnosis was associated with volumetric outcomes. These findings suggest that history of deployment-related mTBI is independently associated with lower volumes in the hippocampus and medical orbitofrontal cortex, and support mTBI as a potential contributing factor to consider in reduced cortical volume in PTSD. These findings are consistent with a prior study that compared individuals with mTBI to those with both mTBI and PTSD [73]. The study reported that mTBI patients displayed only lower entorhinal cortex volumes than individuals with both mTBI and PTSD. Thus, mTBI may have a much stronger relationship to brain volumes than PTSD in combat-exposed veterans.

Finally, the differential impacts of blast-related mTBI and non-blast-related mTBI have been explored by Eierud and colleagues [74]. The authors found significant differences in PTSD Check List–Civilian Version (PCL-C) and Neurobehavioral Symptom Inventory (NSI) scores between blast and non-blast mTBI groups. Cortical thinning was also observed within the blast mTBI group, suggesting that blasts may cause a unique injury pattern related to a reduction in cortical thickness within specific brain regions that could affect symptoms. This study is the first to have found cortical thickness differences between blast and non-blast mTBI groups.

### Fluid Attenuated Inversion Recovery (FLAIR)

Several studies reported the presence of WMHI in mTBI patients. WMHI can be detected through FLAIR MRI and are non-specific findings that can be due axonal/myelin degradation, gliosis, ischemia, and inflammation [75]. Patel et al. found that WMHIs were present in 81% of an mTBI military cohort versus 60% of non-injured military controls [70]. Specifically, a frontal lobe-only distribution of WMHI was more commonly seen in the mTBI cohort. Although the presence of WMHI tends to increase in patients with a history of TBI relative to controls, [76] WMHI are not specific to TBI [77–79].

Generally, prior studies on patients with a history of mTBI reported inconsistent relationships between WMHIs and cognitive outcomes [80–82]. Clark et al. [80] found an interaction between mTBI diagnosis and deep WMHI volume on delayed memory, with mTBI subjects performing worse than controls as deep WMHI volumes increased. In contrast, no relationship was found between deep WMHI volume and executive functioning, nor peri-ventricular WMHI volume and learning/memory/executive functioning. Tate et al. [82] demonstrated that among SMs with a history of mTBI, those with WMHI had worse working memory than those without. However, no group differences were found on tests of processing speed, learning, and memory in this cohort. Spitz et al. [81] studied patients with history of mild to severe TBI and found that those with high frontal WMHI lesion load were slower to complete Trails B (neuropsychological test of visual attention and task switching) than those with low frontal WMHI lesion volume. However, there were no other group differences for other cognitive measures between individuals with high and low total or frontal WMHI volumes.

Other studies found no association between WMHI and self-reported psychological symptoms and cognitive outcomes [80, 82]. Specifically, Berginstrom et al. [83] found no relationship between WMHI and cognition in patients with a history of mild-severe TBI. Likewise, Lippa et al. [84] found no association between whole-brain counts of WMHIs and 36 out of 37 self-report symptomatic and cognitive measures. These studies suggest that WMHIs may not be associated with significant changes in self-reported symptoms or cognitive performance in patients with a history of mTBI. Although methodologic differences may have accounted for the observed differences in prevalence, existing studies emphasize the importance of WMHI findings and acknowledge them as a clinical challenge.

### Deformation morphometry

Deformation morphometric techniques analyze subtle volumetric and shape changes that are often not revealed in traditional volumetric analyses [85, 86]. Several studies demonstrate the association between mTBI and brain morphology. For instance, Tate et al. observed shape differences in the thalamus, nucleus accumbens, and amygdala in a symptomatic cohort of SMs with mTBI when compared to post-deployment controls with orthopedic injuries only [87]. In another study, Tate et al. expanded on this prior study to directly examine the relationship between shape metrics and neuropsychological performance [88]. The study found several significant group-by-cognition relationships with shape metrics across various cognitive domains, including processing speed, memory, and executive function. Higher processing speed was robustly associated with more dilation of caudate surface area among patients with mTBI who reported more than one of the following: LOC, AOC, and/or PTA. The authors concluded that the morphology of subcortical structures is important in cognitive performance following mTBI.

### Diffusion weighted imaging (DWI) and diffusion tensor imaging (DTI)

Diffusion weighted imaging (DWI) is a technique that is sensitive to the movement of water. DWI can detect the movement of water (diffusion) within brain tissue to infer microstructural tissue properties. Diffusion tensor imaging (DTI) is a technique to model the diffusion properties in a voxel to be able to indirectly measure the structural orientation of the water movement and the degree of anisotropy to infer tissue integrity, architecture, and presence of barriers [89, 90]. Specifically, DTI modeling provides a measure of the microstructural integrity of WM fiber tracts, making it a promising biomarker candidate for subtle tissue changes affecting the integrity of the brain’s structural connections following mTBI. Within each voxel, DTI infers specific diffusivity measurements, including the molecular diffusion rate [Mean Diffusivity (MD)], the diffusion direction [Fractional Anisotropy (FA)], the axial (diffusion rate along the main axis), and radial (rate of diffusion in the transverse direction) diffusivity (Table 3) [91, 92].

**Table 3:** Definition and interpretation of DTI metrics in the context of TBI.

DTI metric	Description of measurement	Interpretation in TBI
Fractional Anisotropy (FA)	A scalar value between 0 and 1 that describes the degree of anisotropy of the diffusion process. A value of zero indicates isotropic diffusion (i.e., equal diffusion in all directions).	FA is thought to reflect fiber density, axonal diameter, and myelination in WM. Lower FA has been reported in previous studies on mTBI. Reduced FA is often associated with either increases in AD or RD indicating increased disorganization of WM tracts. While less common than decreased FA, some studies have reported increased FA values in certain brain regions following mTBI, indicative of compensatory mechanisms, gliosis, or changes in water diffusion patterns due to neuroplasticity.
Mean Diffusivity (MD)	A scalar measure of the total diffusion or average mobility of water molecules within a voxel.	MD an inverse measure of the membrane density, is similar for both GM and WM but higher for CSF. MD is sensitive to cellularity, edema, and necrosis. Higher MD has been reported in previous studies on mTBI. Higher MD can be associated with various pathological processes following mTBI, such as cellular swelling, vasogenic edema, and axonal injury.
Axial Diffusivity (AD)	The magnitude of diffusion parallel to fiber tracts or the diffusivity along the main fiber.	Reduced AD may reflect axonal injury, reduced axonal caliber, or less coherent orientation of axons. There is evidence that AD is not influenced by myelin.
Radial Diffusivity (RD)	Coefficient of diffusion perpendicular to the main fiber orientation.	Increased RD is an indication of decreased WM integrity and de- or dys-myelination. Changes in the axonal diameters or density may also influence RD. Increased RD was observed in various brain regions in mTBI patients.

In the context of TBI, altered diffusion properties within WM i.e., dispersed diffusion of water) may be reflective of demyelination and axonal degeneration. Yet, studies of military mTBI generally have yielded varied findings on which WM tracts are affected and whether FA is increased or decreased following injury [93]. Some studies report lower FA after remote mTBI [94, 95], elevated FA [96], or lack of significant mTBI effects on FA [97–99]. These inconsistencies may be due to the variability in mechanism (i.e., different cellular alterations) and etiology of mTBI amid different time points post-injury. For example, in the subacute stage following injury, there would be reduced diffusion along the axial direction (decreased AD) due to axonal injury. In the chronic stage post-injury, there may be increased anisotropy due to predominating neural plasticity and increased coherent processes driving the recovery process [100, 101]. On the other hand, a reduction in diffusivity and an increase in anisotropy in the chronic phase may be attributed to glial hypertrophy/proliferation and the formation of scar tissue, resulting in an increased number or thickness of glial processes and cellular density. Increased diffusivity and decreased anisotropy can also occur as a result of neural repair with microglial phagocytosis [100].

Indeed, the pathophysiological effects of mTBI are highly contingent on the time point post-injury. Thus, in order to gain a deeper understanding of how injury effects develop over time, Donald et al. carried out a longitudinal DTI study. The results revealed a decline in the number of regions of interest (ROIs) with reduced FA at the average of 1 year after the injury. However, at the 5-year follow-up, an increase in the number of regions with reduced FA was observed among SMVs who had experienced concussive blast exposure [102]. The increase in ROIs with reduced FA in the chronic stage may be indicative of microstructural changes underlying the “accelerated brain aging” theory recently reported from chronic, cross-sectional studies of veterans following brain injury [103]. In another recent longitudinal study, Yeh et al. [104] examined quantitative DTI neuroimaging trajectory in SMVs who had sustained an uncomplicated mild, complicated mild, moderate, or severe TBI compared to those who either had sustained an injury without TBI (injured controls) or who had not sustained any injury (non-injured controls). Participants with mTBI presented with WM microstructural changes, mainly in deep central WM over the posterior part of cerebrum, with more spatial involvement in complicated mTBI than in uncomplicated mTBI. Further, uncomplicated mTBI had decreased diffusivity with slightly increased FA compared to controls, suggesting restricted diffusion due to brain repair through neuroplasticity, i.e., astrocytosis with glial processes and scaring.

WM microstructural disruptions have also been explored in co-occurring PTSD and TBI. Lepage et al. discovered FA reductions in patients with both TBI and PTSD compared to TBI alone [105]. Isaac et al. found that lower FA was associated with MDD in veterans with co-occurring PTSD and TBI compared with veterans with MDD alone [106, 107]. Lange et al. [108] observed a significantly higher number of self-reported symptoms on all neurobehavioral measures (e.g., MDD), and lower scores on more than half of the neurocognitive domains (e.g., processing speed) in the mTBI/PTSD-Present group compared to the mTBI/PTSD-Absent and control groups. Yet, no significant group differences in DTI measures were found, with the exception of some regions (i.e., superior longitudinal fascicle and superior thalamic radiation). The authors concluded that there is a strong association between PTSD and poor neuropsychological outcome after mTBI, although there is a lack of association between PTSD and WM integrity, measured by DTI.

### Task-based functional brain imaging

Functional MRI (fMRI) utilizes changes in blood oxygen level-dependent (BOLD) signal to assess neuronal activity and brain function [109, 110]. Changes in BOLD signal within certain brain regions can be attributed to vascular coupling with neuronal activity and thus are an indirect marker of neural activity. Task-based fMRI measures BOLD signal in relation to time-dependent activity of the brain. This enables the identification of specific brain regions that are associated with task performance.

Task-based fMRI has been used to assess cognitive function post-injury. For instance, Sullivan et al. explored cognitive control (via Flanker task) in veterans with a history of blast-related mTBI by assessing both the function and interaction of brain networks [111]. The authors found that behavioral performance did not differ in individuals with and without mTBI, but the neural signature of cognitive control was amplified in the mTBI group. That is, compared to the control group, the mTBI group demonstrated greater deactivation of regions associated with the default mode network (DMN) during the processing of errors. Additionally, error processing in the mTBI group was associated with heightened negative connectivity between the DMN and the dorsal anterior cingulate cortex as well as the dorsolateral prefrontal cortex, regions that are part of the salience and central executive networks. The authors concluded that deactivation of DMN regions and associated increased connectivity with cognitive control regions may act as a compensatory mechanism for successful cognitive control task performance in mTBI, consistent with previous studies [112, 113].

Moreover, Dretsch et al. studied the voluntary regulation of emotion in SMs both with and without chronic mTBI using fMRI and a series of cognitive and psychological health measures [114]. Subjects were instructed to maintain (passively view), enhance (i.e., make the negative feelings toward images more extreme), and suppress emotions associated with negative and neutral visual stimuli (military-relevant images). The mTBI group presented with significantly greater clinical symptoms, but only a mild decrease in attention. Specifically, the mTBI group presented with greater activation in the precentral gyrus, postcentral gyrus, inferior parietal lobe, insula, and superior temporal gyrus. When controlling for PTSD symptoms, a differential neural activation pattern was found only during the enhance condition in mTBI subjects compared to controls. Increased activation of the frontal and limbic regions was associated with the effect of PTSD symptoms during the enhance condition. Thus, hyper-activation of brain regions in the mTBI group during the enhance condition may reflect vigilance towards negative contextual stimuli and/or suboptimal allocation of energy to regulate emotions. The findings suggest that, compared to deployment-exposed controls, symptomatic soldiers with combat-related mTBI have altered neural activity patterns during the voluntary regulation of emotions. Altogether, these studies suggest that mTBI is associated with altered brain activity that may include compensatory neural activation to recruit more neural resources for the same task. Further research is needed to understand whether enhanced activation reflects compensatory processes or is associated with other unknown processes in the injured brain.

### Resting-state functional connectivity

Resting-state fMRI (rs-fMRI) or resting-state functional connectivity (rs-FC) measures the temporal correlation of spontaneous BOLD signal among spatially distributed brain regions. The correlated activities of these brain regions are assumed to form functional networks. In contrast to task-based fMRI, rs-fMRI observes brain activity in the absence of a task performance or stimulation. The two most common techniques for analyzing rs-FC are seed-based correlations and independent components analysis (ICA). In the seed-based technique, signal is extracted from a specific ROI, and a map is created by computing the correlation between this extracted signal and all other brain voxels [115, 116]. Conversely, using a mathematical algorithm, ICA observes all voxels and identifies distinct brain networks that are correlated in their spontaneous fluctuations but also spatially independent [117–119].

Recently, Sheth and colleagues used a seed-based approach to study the rs-FC of the anterior cingulate cortex (ACC) in veterans with mTBI, given the region’s critical role in emotion regulation and executive function [120]. The study found increased connectivity of the left and right ACC with brain regions including middle and posterior cingulate regions, precuneus, and occipital regions in the mTBI compared to the non-TBI group. These findings suggest the presence of hyperconnectivity in veterans with mTBI and are consistent with previous studies of recently concussed athletes showing ACC hyperconnectivity. The authors concluded that enhanced top-down control of attention networks via ACC hypoconnectivity may be necessary to compensate for the microstructural damage following mTBI.

Similarly, Pagulayan et al. analyzed the effect of blast-related mTBI on the working memory functional connectivity system using rs-fMRI [121]. Reduced working memory is frequently reported by veterans with a history of blast-related mTBI but can be difficult to quantify through neuropsychological measures. The study observed no group differences in neuropsychological measures of working memory. However, widespread hyperconnectivity from the frontal seed regions in the mTBI group relative to the deployed control group was observed. Further, within the mTBI group, but not the control group, better working memory performance was associated with increased functional connectivity from frontal seed regions to multiple brain regions, including cerebellar components of the working memory network. Consistent with prior studies [122–124], the authors concluded that long-term alterations in the functional connectivity of the working memory network following blast-related mTBI may reflect a compensatory change for properly performing a working memory task and that hyperconnectivity is a common post-TBI neural response [125].

Patterns of rs-FC have also been compared between those with mTBI versus PTSD. Philippi and colleagues examined whether there are differences in rs-FC of major cortical networks between SMs with mTBI without PTSD, PTSD without mTBI, and orthopedically injured controls (OI) [126]. Reduced rs-FC for DMN and frontoparietal regions was observed in both mTBI and PTSD groups, compared with OI controls, with the PTSD group showing more diminished connectivity. Yet, rs-FC with the middle frontal gyrus of the FPN was increased in mTBI, but decreased in PTSD. The authors concluded that the observed opposite patterns of connectivity of the lateral prefrontal cortex highlight a potential biomarker that could be used to differentiate between military-related PTSD and mTBI.

### Graph theoretical approaches

Both brain function and structure can be characterized using graph theoretical approaches that aim to characterize the spatial relations between brain regions (i.e., topology) at the global (i.e., whole-brain, large-scale networks) or nodal level (i.e., individual brain region as part of the network) [127, 128]. Essentially, brain networks can be seen as graphs composed of nodes (i.e., distinct brain regions) that are linked by edges, which can be either structural (i.e., WM fiber tracts) or functional (correlated activity between regions). Graph theoretical measurements are categorized into either network segregation (i.e., clustering coefficient, modularity) or network integration (i.e., global efficiency or characteristic path length) (Table 4) [129]. A detailed explanation of graph theory mathematical equations can be found in the referenced articles [127, 130–133]. Generally, healthy brains consist of small-world network topologies that balance both segregation and integration for coordinated information processing. Thus, small-world networks are highly clustered (a characteristic of lattice networks) but possess relatively short characteristic path lengths (a property of random networks) [134].

**Table 4:** Graph theory metrics used to study TBI.

Graph Theory Metrics	Description of measurement	Interpretation in TBI
Small-World Network	Mathematical graph in which most nodes are not neighbors of one another, but the neighbors of any given node are likely to be neighbors of each other. Most nodes can be reached from every other node by a small number of paths. Both anatomical connections in the brain and the synchronization networks of cortical neurons exhibit small-world topology. Small-worldness of neural networks is associated with efficient communication.	There is evidence that key brain networks associated with cognitive function have reduced small-world topology after TBI. This may be due to diffuse WM damage, and reduced small-worldness may be associated with cognitive impairment after TBI.
*Network Segregation*		
Clustering Coefficient	A measure of the degree to which interconnected nodes in a graph tend to cluster together. Clustering coefficient reflects the number of connections that exist between the nearest neighbors of a node as a proportion of the maximum number of possible connections. It is the difference in mean within- versus between- community connections, relative to the mean within-community connections of a network.	Higher clustering coefficient is observed after TBI. This finding indicates that TBI patients have network graphs with increased functional segregation. Clustering coefficient was found to be associated with processing speed in TBI patients.
Modularity	A measure that quantifies the degree to which functional brain networks are divided into distinct subnetworks.	TBI patients present with disrupted modular organization of the whole brain (i.e., increased modularity and altered within-module connectivity, relative to healthy individuals).
*Network Integration*		
Global Efficiency	A measure that indicates how effectively information is integrated across the entirety of the brain network. It is defined as the inverse of the average characteristic path length between all nodes in the network.	TBI patients present with decreased global efficiency of brain networks. Significant correlations between switching performance and global efficiency within TBI subjects were found. TBI patients may have a weaker globally integrated structural brain network, resulting in a limited capacity to integrate information across brain regions. Reduced global efficiency is likely due to disrupted diffuse white matter (axonal) integrity as indicated by its significant negative correlation with the decreased FA.
Characteristic Path Length	A measure indicating the efficiency of information or mass transport on a network. It is the average number of steps along the shortest paths for all possible pairs of network nodes. Short average path length facilitates quick transfer of information and reduces costs.	Longer characteristic path length was found in TBI patients compared to healthy controls. This indicates that there are a greater number of steps between any two nodes on average in the TBI network compared to the HC network. Longer characteristic path length correlated with worse performance on verbal learning task as well as executive dysfunction in TBI patients.

In recent years, graph theoretical approaches have been applied to FC alterations associated with TBI [135, 136]. These studies indicate that TBI is associated with network hyperconnectivity as demonstrated by increased density and clustering coefficient, and suboptimal global integration [135, 137]. Thus, mTBI may lead to reduced connectivity and network efficiency with increased path lengths [138], clustering coefficients, and aberrant modularity [139, 140]. Further, small-world network topology was disrupted in participants with PTSD and mTBI [136, 138, 141, 142]. At the nodal level, disruptions in the frontal-limbic network [143, 144] were observed in both mTBI and PTSD, implicating the cingulate cortex as a potential basis for shared symptomatology [138].

Graph theory analysis of rs-fMRI has also been conducted in longitudinal mTBI datasets [140, 145, 146]. Messé et al. discovered a notable decrease in network modularity among individuals with PCS who had experienced mTBI compared to those without such symptoms [140]. Dall’Acqua et al. observed functional hypoconnectivity in the DMN of mTBI patients relative to controls during the acute phase of recovery, although this hypoconnectivity normalized over the course of a year [145]. Recently, Boroda et al. found that brain networks were less clustered and more modular in individuals with mTBI [146]. Over time, however mTBI networks became more densely connected as observed by increased clustering and reduced modularity, while no changes across time were observed in healthy controls. Overall, these studies show that brain networks remain plastic following injury and undergo significant changes in network topology over time.

Graph theoretical approaches have also been applied to structural morphology [130, 143, 144, 147]. Comparatively, structural network properties may be less sensitive to differences in cognitive state or task proficiency but may provide a more robust depiction of long-term alterations in brain function as a consequence of Hebbian plasticity [148]. Recently, Proessl et al. explored cortical thickness-based structural covariance networks of SMs with PTSD, mTBI, and mTBI-PTSD compared to healthy SMs [149]. Higher levels of arousal, stress, anxiety, and depression were observed in all clinical groups compared with the controls. Nodal analysis revealed altered path lengths and closeness centrality in fronto-limbic areas in mTBI-PTSD. The authors concluded that mTBI and PTSD may be associated with distinct pathophysiological manifestations in structural brain networks.

## Promising frontiers in neuroimaging research for military-related mTBI

### Leveraging new diffusion MRI techniques and a multi-modal neuroimaging approach

Newer diffusion techniques have evolved in recent years and show more promise as a potential biomarker for mTBI. FA in standard DTI falls short of adequately defining the tissue microstructure and thus misses subtle changes in areas with multiple WM tracts running in different orientations. Accordingly, taking advantage of the kurtosis tensor can help to add the non-Gaussian diffusion components to the model and better define the microarchitecture in the voxel [150, 151]. Diffusion kurtosis imaging (DKI) involves an expansion of the diffusion sequence itself, adding a collection of additional b-values, which indicate the strength or intensity of the diffusion-sensitizing gradients applied, thereby improving sensitivity and estimation of water movement. The addition of multiple b-values acquired in a framework of multiple q-shells allows for the collection of several measurements in a radial decay function that increase spatial diffusion signal so that angular accuracy is improved. This is referred to as diffusion multi-shell imaging [152]. Diffusion multi-shell techniques hold promise in detecting microstructural abnormalities in WM tracts from clinical scanners, as conventional sequences still cannot show such microstructural damages from mTBI [153]. Recently, Chung, et al. acquired multi-shell diffusion with 5 b-values and multiple diffusion directions. Using denoising algorithms paired with a WM integrity metric that evaluates both intra- and extra-axonal environments, along with DTI and DKI metrics, they were able to measure decreased intra-axonal diffusivity along the axons [154].

**Fig. 4:**
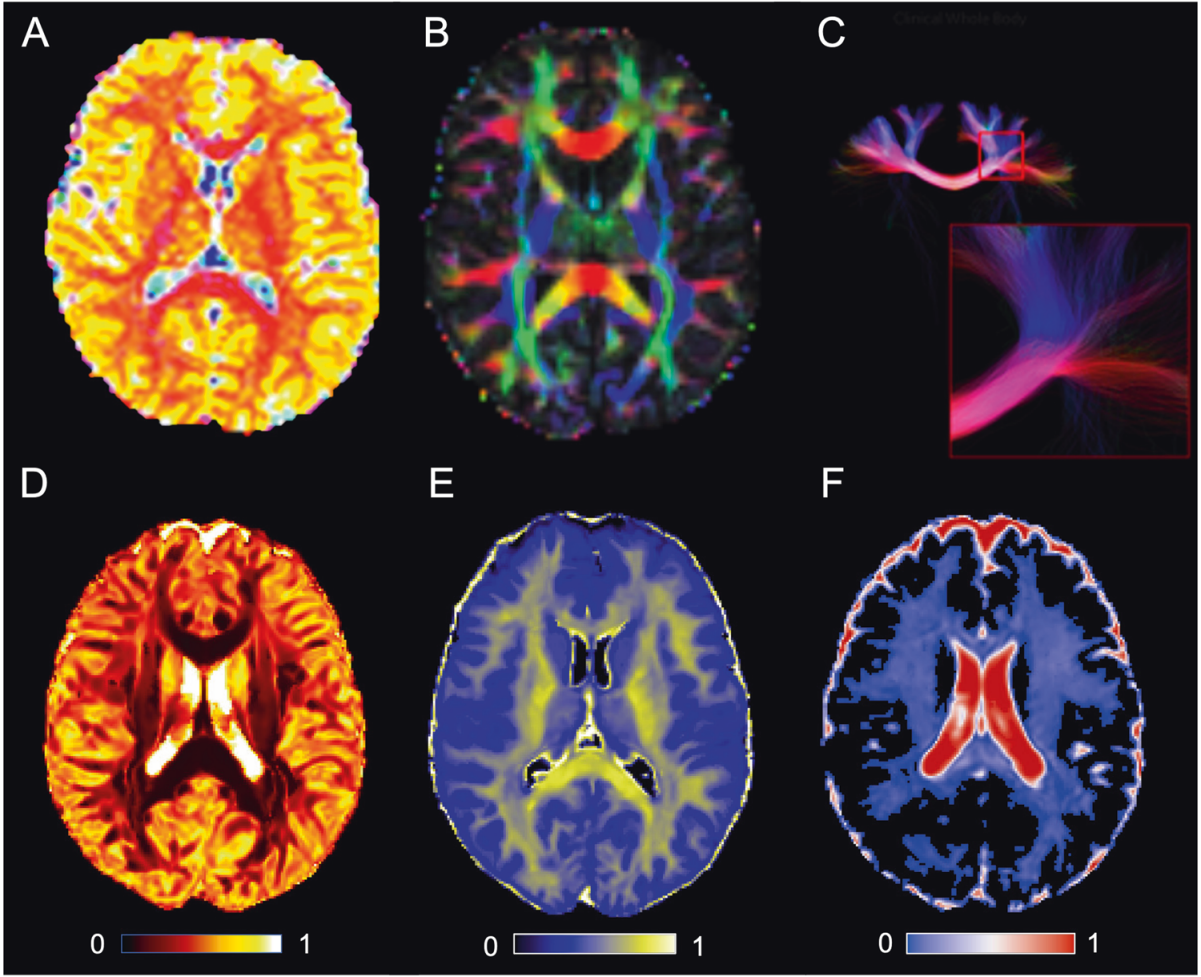
Diffusion multi-shell MRI techniques. Standard diffusion techniques utilize a single b-value to measure water movement in the brain along the white matter tracts, usually underestimating the restriction in the voxel. Multi-shell techniques utilize multiple b-values and can improve the ability to detect features of the cellular environment and better estimate the white fiber tracts within a voxel. **A** shows a multi-shell axial image acquired at 3 T. **B** shows an FA map from a 3 T GE MR 750 scanner. **C** is a zoomed in tractography view of the centrum semioval from the same patient showing the white matter pathways that can be seen with conventional 3 T MR scanner using the multi-shell diffusion technique. Note the complex fiber angles in the close-up view. **D** shows a NODDI orientation dispersion index (ODI) map, with lighter colors representing values closer to 1. **E** shows a NODDI intra-cellular volume fraction (ICVF) map, with lighter colors representing values closer to 1. **F** shows a NODDI isometric volume fraction (ISOVF) map, with red colors representing values closer to 1.

Yet, the metrics derived from the DTI and DKI lack structural specificity. For this reason, a neurite orientation dispersion and density imaging (NODDI) model was created to offer more specific indices of tissue microstructure [155]. The NODDI model uses diffusion metrics representing tissue characteristics, including the orientation dispersion index (ODI) indicating dispersion or variation in the orientations of neurites (axons and dendrites) within a voxel, the intra-cellular volume fraction (ICVF) indicating the fraction of the voxel occupied by intra-cellular structures, primarily neurites, and intra-cellular volume fraction (ISOVF) indicating the volume of intra-cellular signal relative to the total volume of the voxel. A recent study on civilian patients used NODDI to identify longitudinal WM changes of declining neurite density after mTBI, suggesting axonal degeneration from diffuse axonal injury [156]. The authors concluded that NODDI metrics are more sensitive/specific biomarkers than DTI for WM microstructural changes following mTBI. Together, DKI and NODDI show promise in mTBI research (Fig. 4), and further research is needed in military populations as described in Fig. 1.

A potential alternative for enhancing neuroimaging capabilities lies in the development of high-gradient technology MRI systems, which show promise in improving the visualization of microstructures, particularly in diffusion techniques [157–159]. Enhancements in diffusion, spatial, and angular resolution can be achieved through high gradient amplitude and high slew rates. By increasing the gradient strength in diffusion MRI, it becomes possible to obtain higher diffusion values without compromising the signal-to-noise ratios. This advancement in gradient strength has the potential to improve the ability to resolve microstructure, consequently facilitating superior visualization of fiber orientation crossings [160]. In a comparative study between a conventional 3T scanner and a high-gradient head-only system, it was observed that the utilization of the higher gradient system allowed for shorter echo times (TEs) and reduced diffusion encoding, resulting in decreased echo spacing. This reduction in echo spacing led to improved image quality by mitigating blurring and distortion, as compared to the conventional 3T MRI setup [161, 162]. These high-gradient systems also allow for more advanced pulse sequences to be developed such as oscillating gradient spin echo (OGSE) diffusion [163]. The stronger gradients can achieve high b-value and frequency simultaneously to increase diffusivity. In the initial investigation involving acute TBI patients, the utilization of OGSE and multi-shell DTI techniques revealed WM abnormalities in time-dependent parallel diffusivity and kurtosis maps. In contrast, no abnormalities were detected using conventional techniques or the conventional 3T MRI scanner [164].

Finally, employing a multi-modal MRI approach can help obtain a more comprehensive assessment of various aspects of brain structure, function, and connectivity following mTBI as well as aid in the identification of potential biomarkers. For instance, one study describes distinct multidimensional MRI signature, derived from a combination of DWI, T1w imaging, T2w imaging, that is associated with microscopic tissue alterations due to diffuse axonal injury. Multi-modal imaging approaches in conjunction with histological techniques advance the neuroimaging field closer towards non-invasive quantitative ‘histology’ that may help clinicians detect and visualize microscopic lesions in the brain [165]. Further, recognizing the characteristic multidimensional MRI signature of various types of brain injuries can enhance the ability to identify and diagnose mTBI accurately, while developing targeted and effective treatment strategies for individuals.

### Using big data to predict brain age, unravel genetic influences, and account for comorbid confounds

Brain age studies are warranted as there is growing evidence on the effect of mTBI on accelerated brain aging from chronic pro-inflammatory microglial profiles post-injury that induce immune cells for dysfunctional responses and neurodegeneration [166, 167]. For instance, one study demonstrated that exposure to TBI lowers the age at which individuals experience cognitive decline, regardless of whether they have Alzheimer’s disease (AD) or non-AD conditions [168]. This information has implications for clinical practice and emphasizes the significance of recognizing TBI history when assessing cognitive function and managing cognitive decline in patients. “Brain age” can be estimated by comparing an individual’s brain scan to a model generated from a large dataset of healthy participants [169, 170].

Recently, Dennis et al. used structural MRI data to examine brain aging in a large, longitudinal sample of SMVs with a history of mTBI [171]. Advanced brain age was observed in males, but not females, with a history of deployment-related mTBI compared to those without mTBI. This association was also present only for deployment-related and blast-related mTBI, but not for non-deployment mTBI. The authors speculated that the reason deployment-related mTBI was associated with brain age could be due to multiple mechanisms of mTBI and secondary effects of the deployment setting such as added stress when the injury was sustained [172]. In follow-up analyses of the male participants, advanced brain age was found to be associated with severity of PTSD and MDD symptoms, and alcohol misuse. These findings support the notion that mTBI can have long-lasting effects on neuropsychiatric outcomes and age-related neurodegeneration [103, 173].

Another area of research pertains to the analysis of large genomic datasets coupled with multi-modal neuroimaging [174]. For instance, the DoD Alzheimer’s Disease Neuroimaging Initiative (DOD-ADNI) study collects clinical, multi-modal neuroimaging, genetics, and biospecimen biomarkers from veterans with a history of TBI [175]. The goal of the study is to examine the connections between TBI and PTSD on brain aging and neurodegeneration. Recently, Clark et al. analyzed the dataset to determine higher CSF tau in veterans with a history of TBI [176]. Yet, additional research is necessary to elucidate the connections between various biomarkers and their ability to predict outcomes (Fig. 1). Studies should also make the effort to consider other comorbidities and potential confounding factors of mTBI subjects, including those related to pain, substance abuse, health service utilization, cardiometabolic risk factors, sex, and ethnicity, and clarify whether such factors have been included in statistical corrections or affect neuroimaging results [177–179]. Importantly, big data analysis of military-related mTBI will enable a personalized medicine approach and the clinical translation of advanced neuroimaging techniques [174, 180].

### Combining blood-based and neuroimaging biomarkers

The risk for neurodegeneration following mTBI highlights the importance of combining neuroimaging and blood-based biomarker analyses. Peripheral blood biomarkers sampling is relatively non-invasive, as acquiring blood samples from patients is a more accepted clinical practice than CSF acquisition, and can provide substantial information regarding specific neurological injury processes of the brain and neuroendocrine-immune signaling processes between the CNS and periphery [181]. Recently, Lippa et al. [182] examined the relationship between plasma tau and Aβ42, neuropsychological functioning, and WM integrity as determined through DTI metrics in SMs with and without a history of uncomplicated mild, complicated mild, or moderate/severe/or penetrating TBI. No association was found between the plasma biomarkers and neurocognitive performance in any of the TBI groups. However, higher tau and Aβ42 were related to higher FA and lower MD, RD, and AD in patients with a history of moderate, severe, or penetrating TBI, although this association was not significant after correction for multiple comparisons. The authors concluded that future work should aim to analyze other blood biomarkers, such as phosphorylated tau instead of total tau and exosomal tau. A more detailed review of potential blood-based biomarkers for mTBI can be found in a recent review by Lippa and colleagues [183]. Overall, further research is needed to identify promising blood-based biomarkers and their connection with neuroimaging correlates for military-related mTBI [184] (Fig. 1).

### Developing neuroimaging techniques to study glymphatic function

Sleep disturbances are one of the most common problems after mTBI [185], and it is becoming increasingly important to understand how glymphatic function is altered following injury. Post-mTBI sleep disturbances impair the recovery process and are associated with persistent neuropsychiatric symptoms [186]. Moreover, post-mTBI symptoms can further disrupt sleep, creating a vicious cycle. The mechanisms underlying this bidirectional relationship remain largely unknown but are discussed in a recent review article [187]. It has been hypothesized that mTBI may lead to glymphatic dysfunction, thus impairing the brain’s ability to clear intestinal solutes and waste.

Glymphatic dysfunction may be inferred by enlarged perivascular spaces (PVSs) detected through MRI. For instance, mTBI in SMVs was associated with an increase in PVS burden, which may indicate waste clearance dysfunction and persistent post-concussive symptoms [188]. Future research should aim to enhance imaging techniques to understand glymphatic function. One analysis technique characterizes diffusion along the perivascular space (DTI-ALPS) [189]. The DTI-ALPS index evaluates the motion of water molecules in the direction of the perivascular space by measuring diffusivity using the diffusion tensor method [189, 190]. This analysis technique has been utilized to discover glymphatic dysfunction in several neurodegenerative diseases, including Alzheimer’s disease [189], Parkinson’s disease [191], and normal pressure hydrocephalus [192]. However, no study to date used DTI-ALPS to characterize glymphatic function after military-related mTBI, and further investigation is warranted.

## Conclusion

Identifying neuroimaging biomarkers that can reliably diagnose mTBI and predict adverse outcomes and recovery of patients remains an ongoing research pursuit. Although mTBI is usually not associated with overt clinical imaging findings, a plethora of studies using advanced neuroimaging techniques have found important differences in imaging metrics and outcomes. This review sought to provide an overview of such advanced neuroimaging techniques, while highlighting the most recent neuroimaging findings focused on military populations. Challenges in mTBI research that remain to be addressed include the lack of standard advanced neuroimaging guidelines for diagnosing mTBI, heterogenous imaging acquisition and analysis methods across study sites, and ambiguity of military-related mTBI mechanisms, timing, and characteristics in deployment settings. New frontiers in neuroimaging research should aim to identify novel imaging techniques to accurately depict tissue microstructure and glymphatic function, explore multi-modal approaches using blood-based and genetic biomarkers, and promote big data analyses to predict mTBI outcomes.

### Disclaimer

The opinions and assertions expressed herein are those of the author(s) and do not reflect the official policy or position of the Uniformed Services University or the Department of Defense.
